# Supplementary material for: RNA Helicase DDX3 Interacts with the Capsid Protein of Hepatitis E Virus and Plays a Vital Role in the Viral Replication
Source: Pathogens. 2025 Feb 10;14(2):177. doi: 10.3390/pathogens14020177 (PMC11858535; doi:10.3390/pathogens14020177)
Supplement: Supplementary file 1 [file pathogens-14-00177-s001.zip › OriginalBlottingImages.pdf]

Figure S1

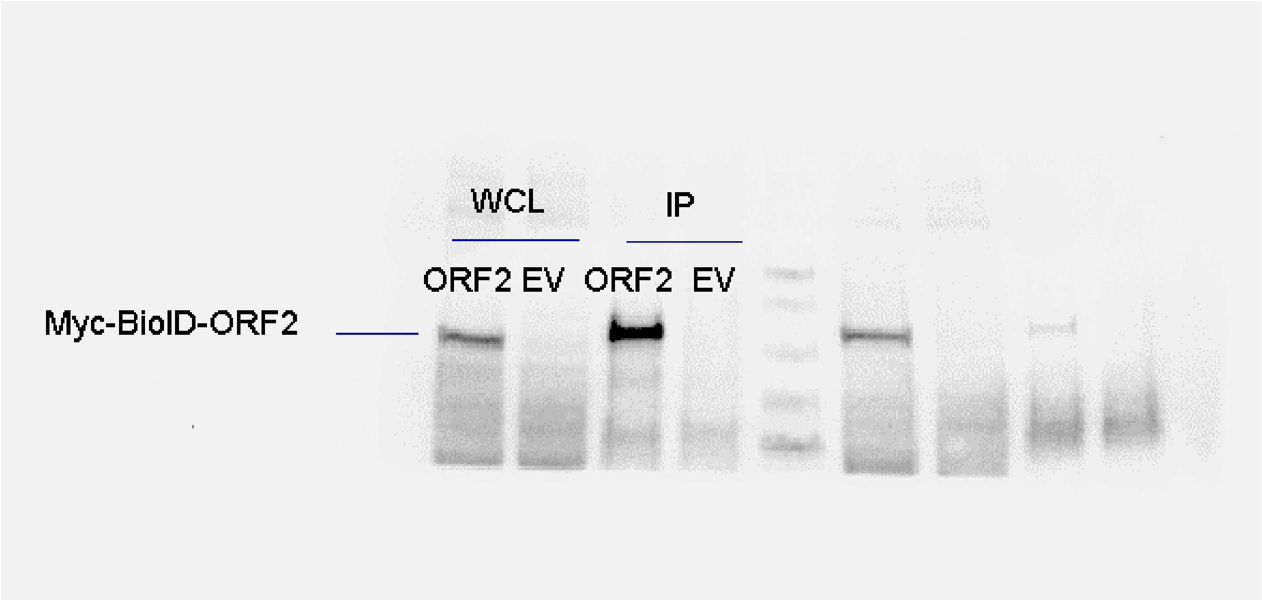

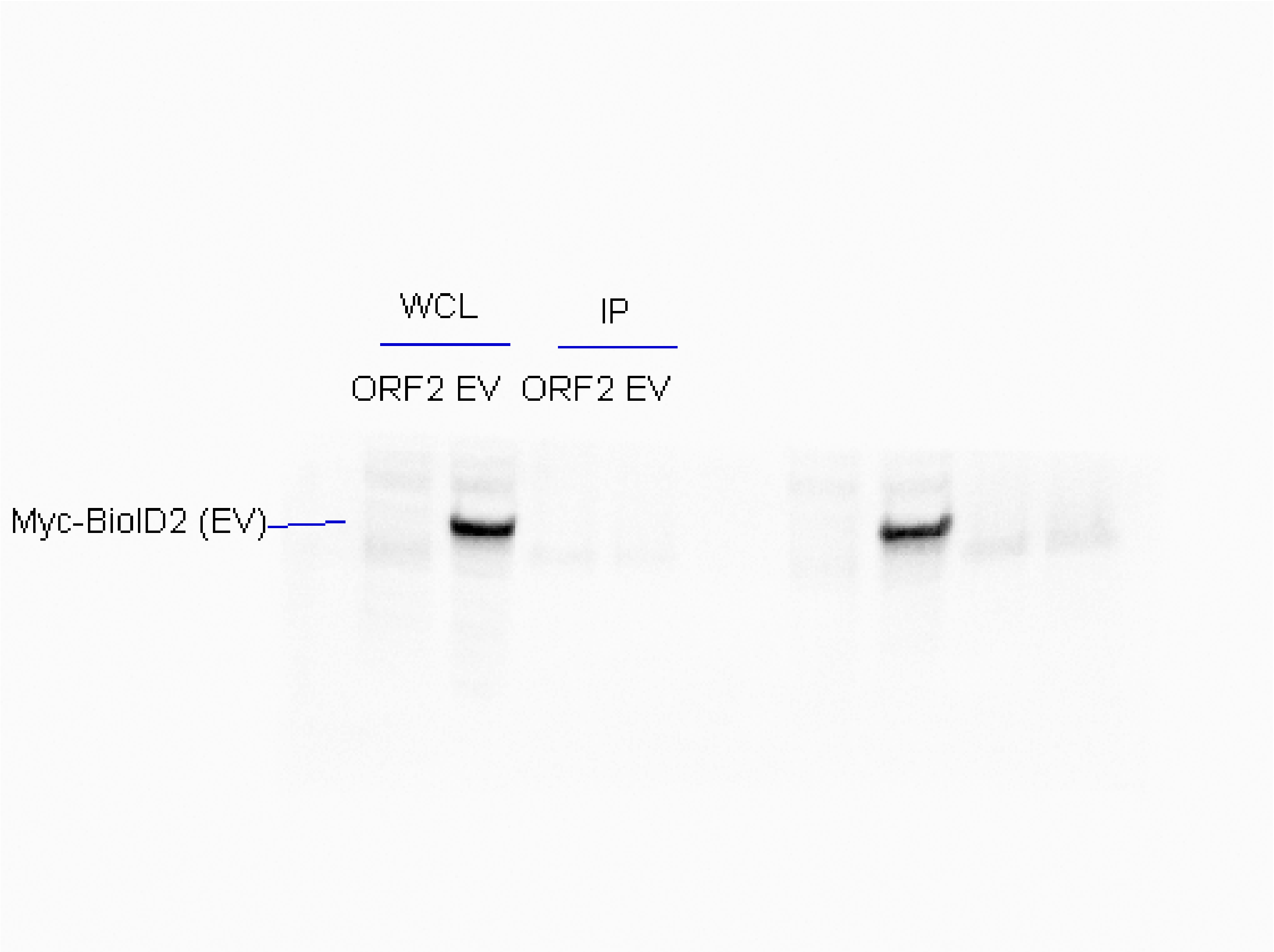

WCL: Whole cell lysate. It is input for IP.

Figure S2

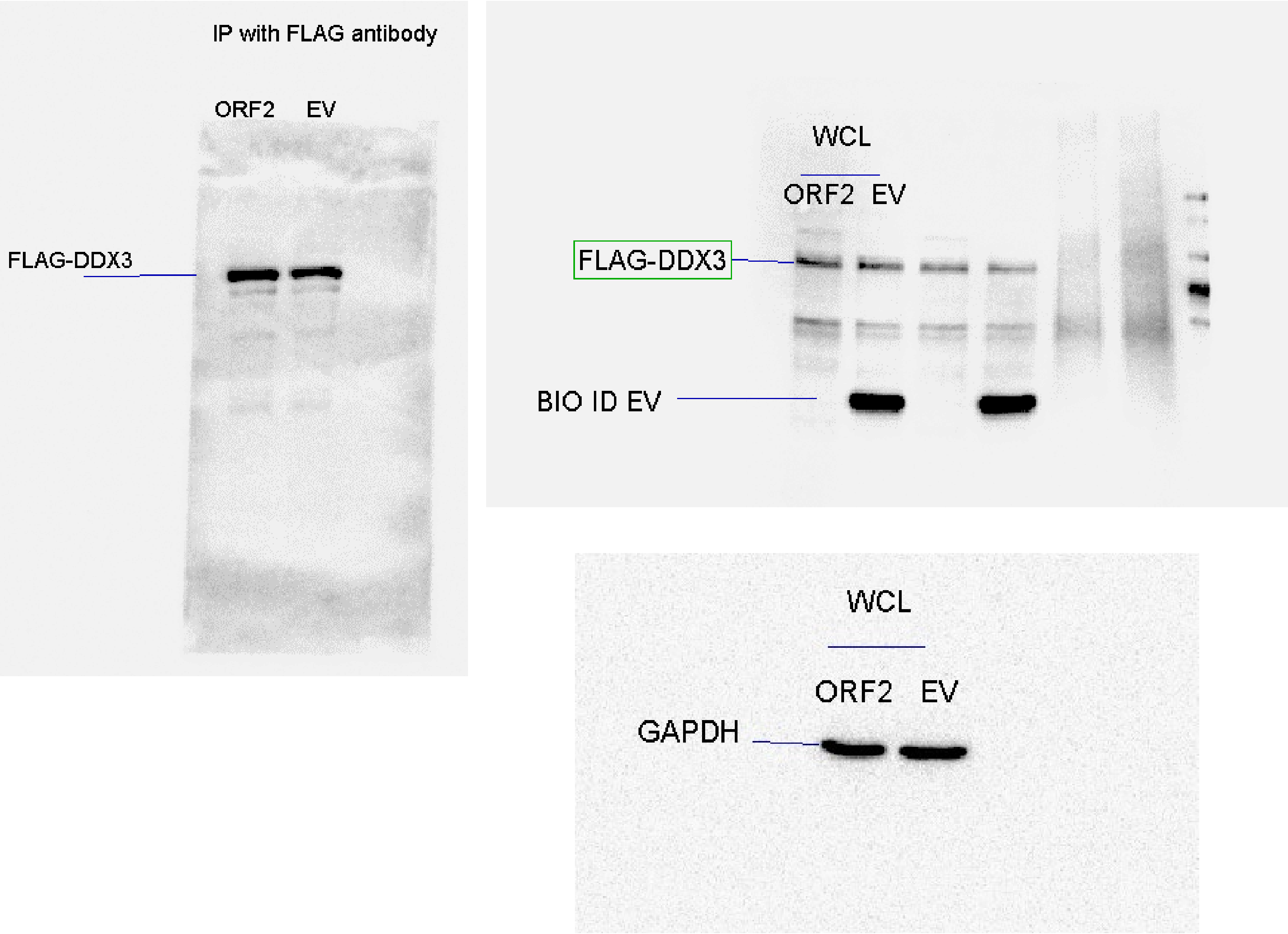
IP with FLAG antibody
ORF2 EV
FLAG-DDX3
WCL
ORF2 EV
FLAG-DDX3
BIO ID EV
WCL
ORF2 EV
GAPDH

Figure S3

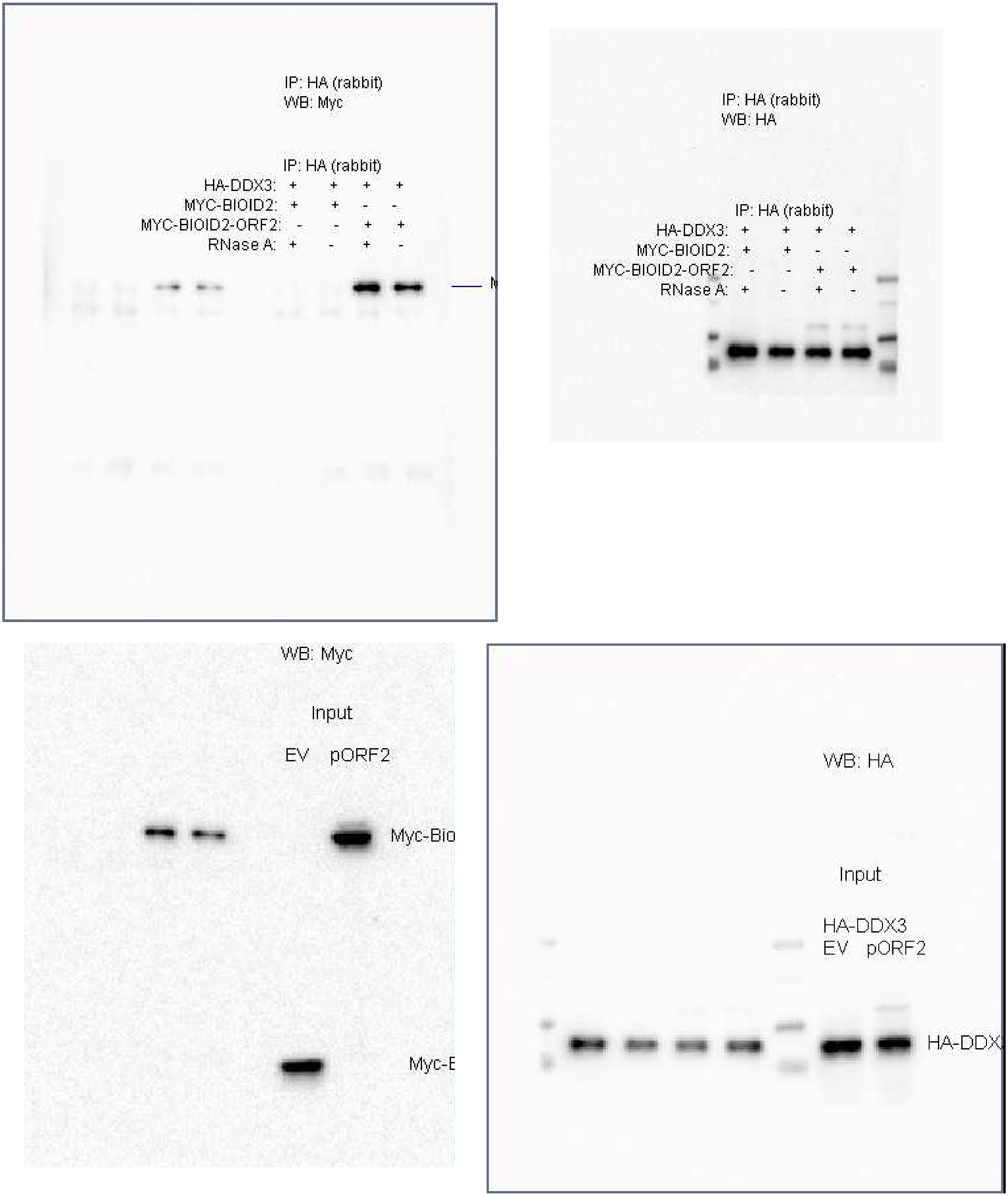
IP: HA (rabbit)
WB: Myc
IP: HA (rabbit)
HA-DDX3: + + + +
MYC-BIOID2: + + - -
MYC-BIOID2-ORF2: - - + +
RNase A: + - + -
IP: HA (rabbit)
WB: HA
IP: HA (rabbit)
HA-DDX3: + + + +
MYC-BIOID2: + + - -
MYC-BIOID2-ORF2: - - + +
RNase A: + - + -
WB: Myc
Input
EV pORF2
Myc-Bio
Myc-E
WB: HA
Input
HA-DDX3
EV pORF2
HA-DDX

Figure S4

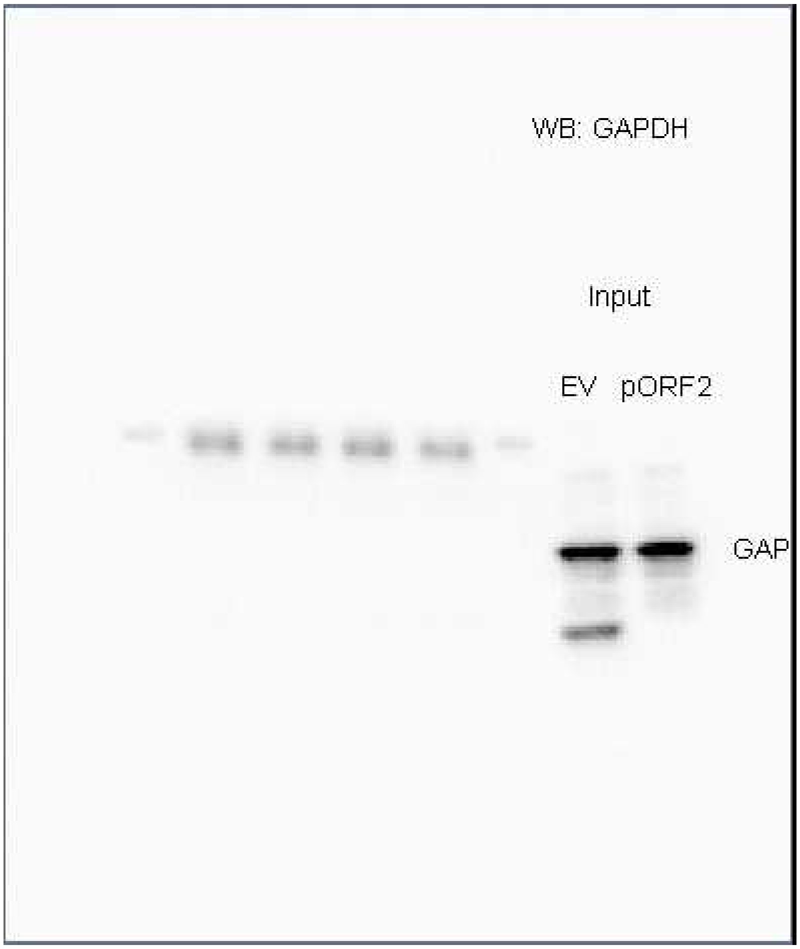

Figure S5

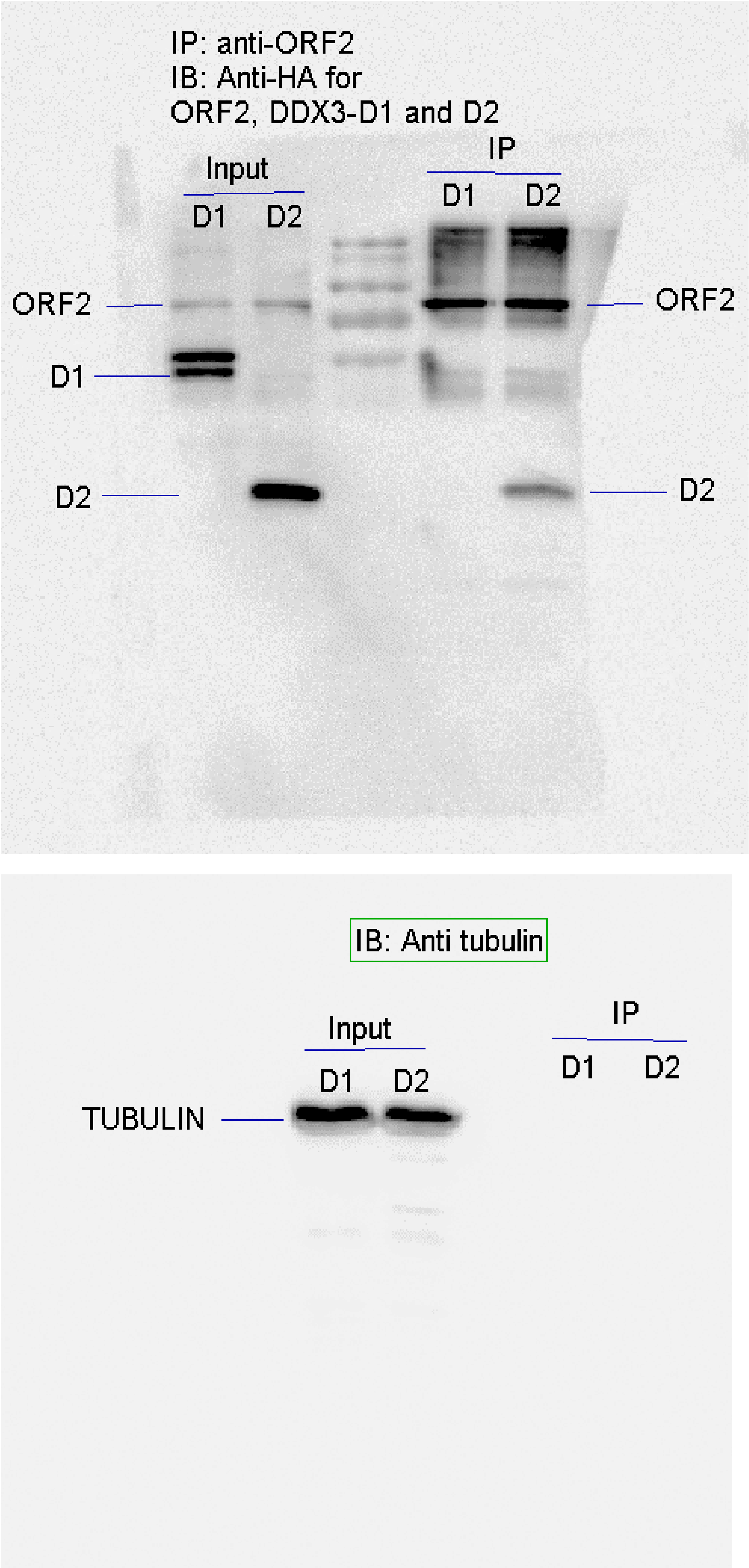
IP: anti-ORF2
IB: Anti-HA for
ORF2, DDX3-D1 and D2
Input
D1
D2
IP
D1
D2
ORF2
D1
D2
ORF2
D2
IB: Anti tubulin
Input
D1
D2
IP
D1
D2
TUBULIN

Figure S6

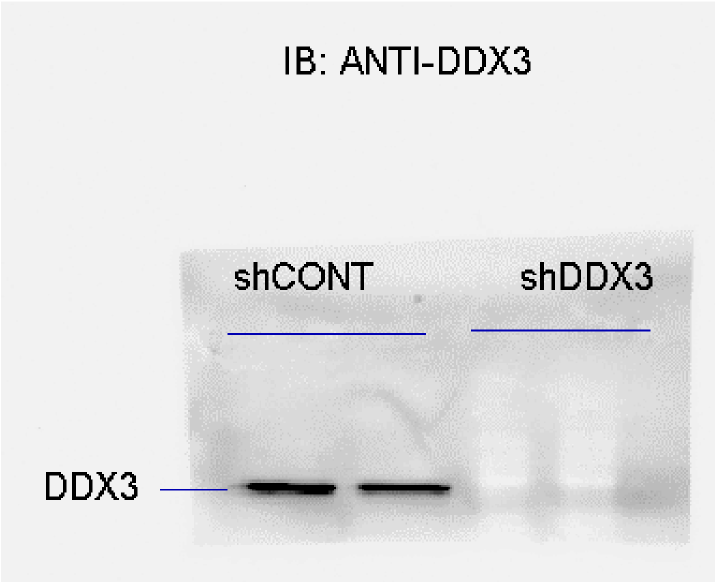

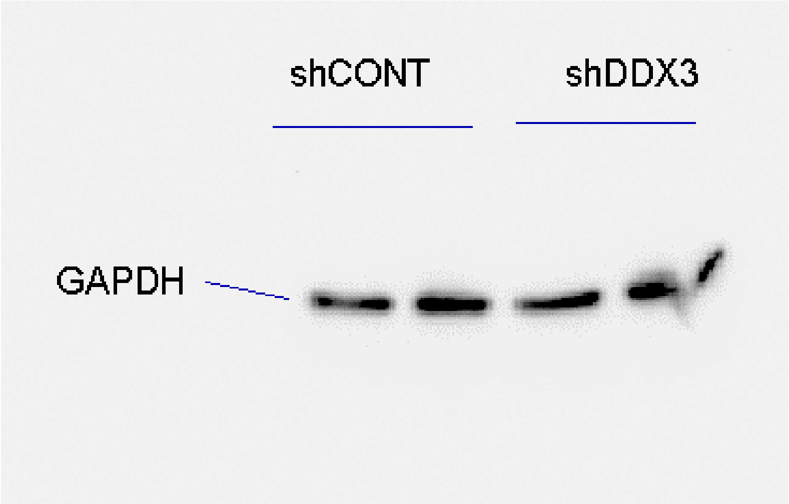

Figure S7

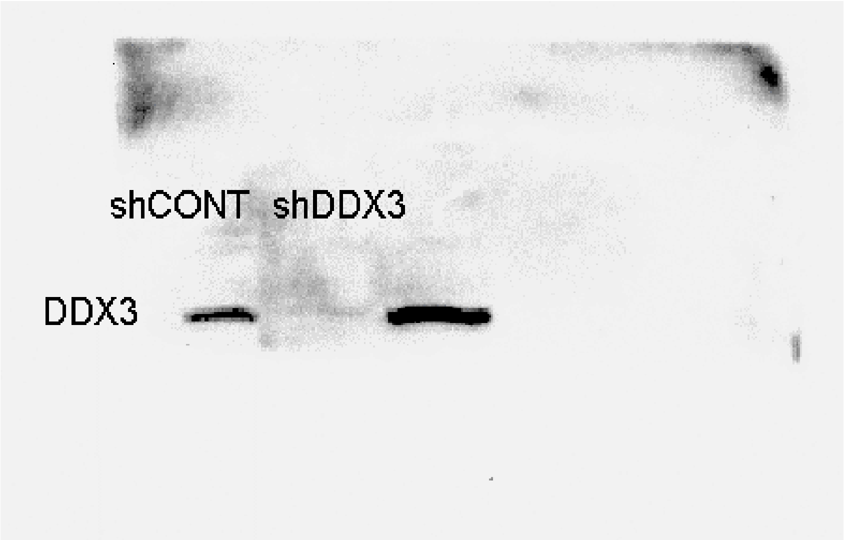

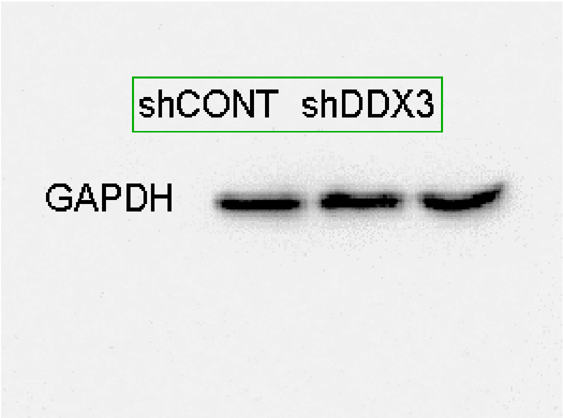

Figure 5D

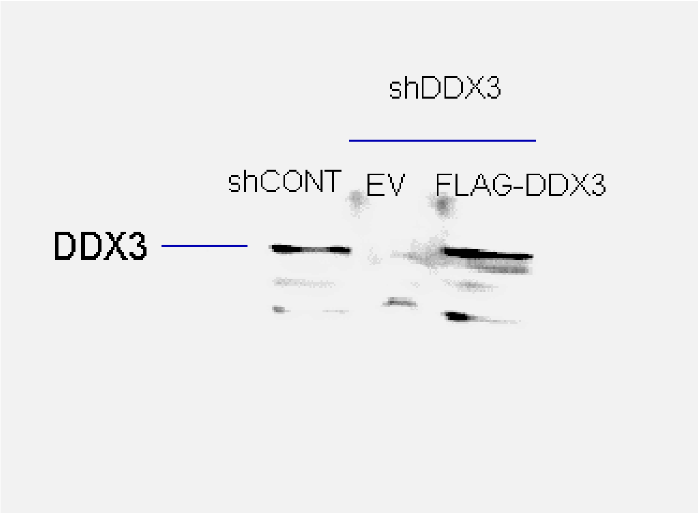

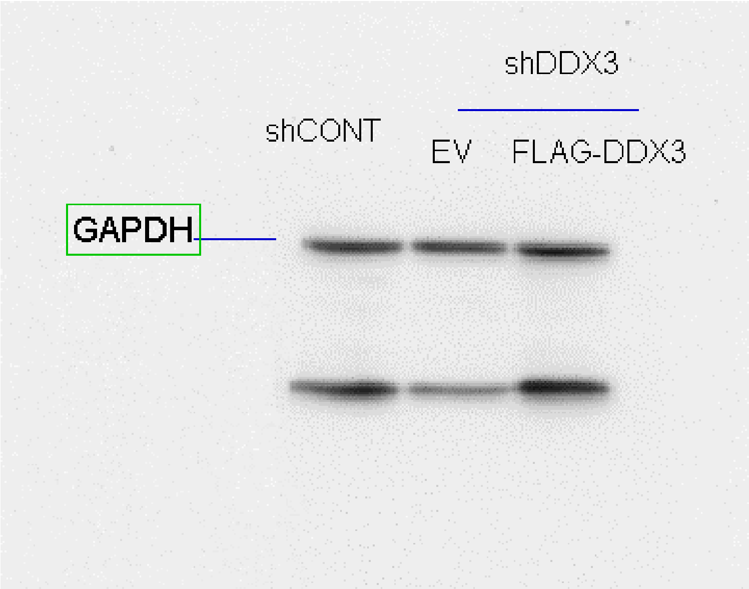

Figure S8

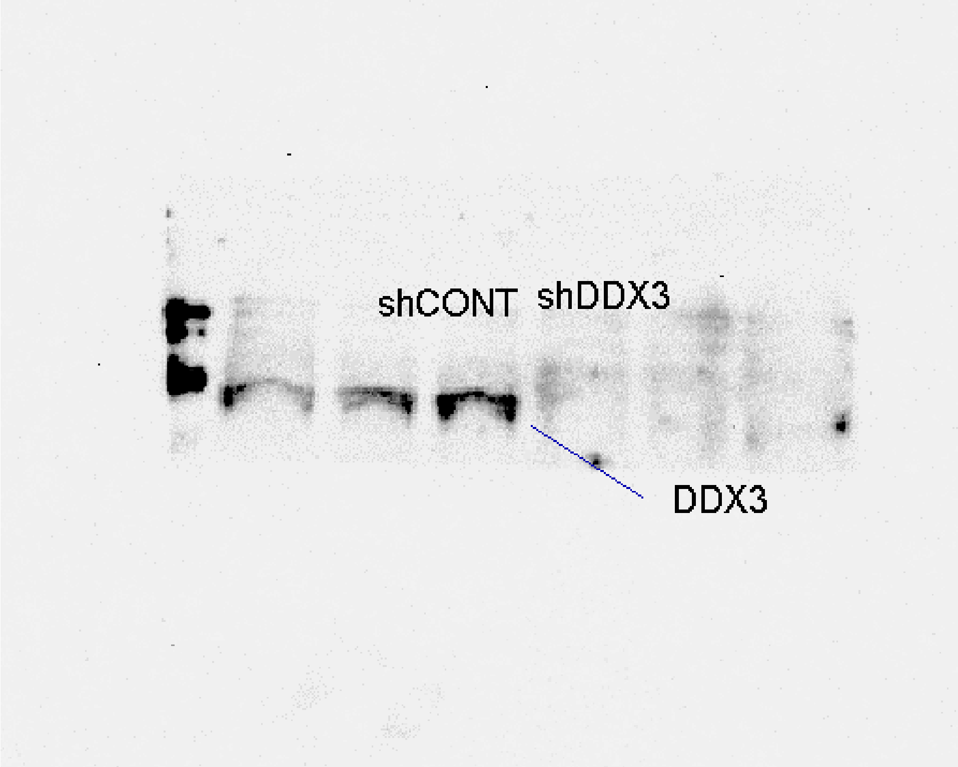

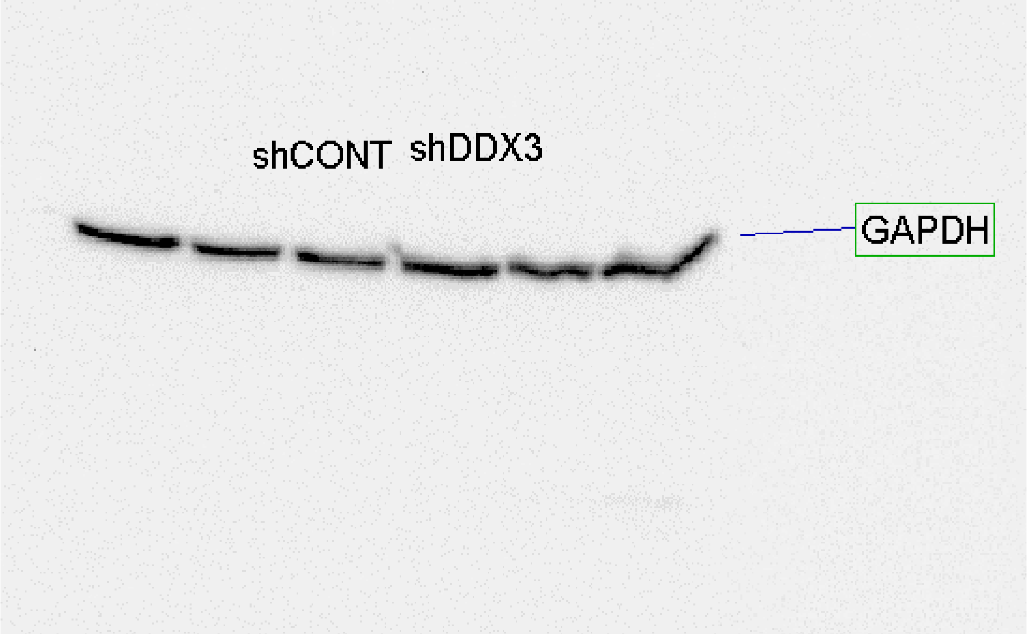

Figure S9

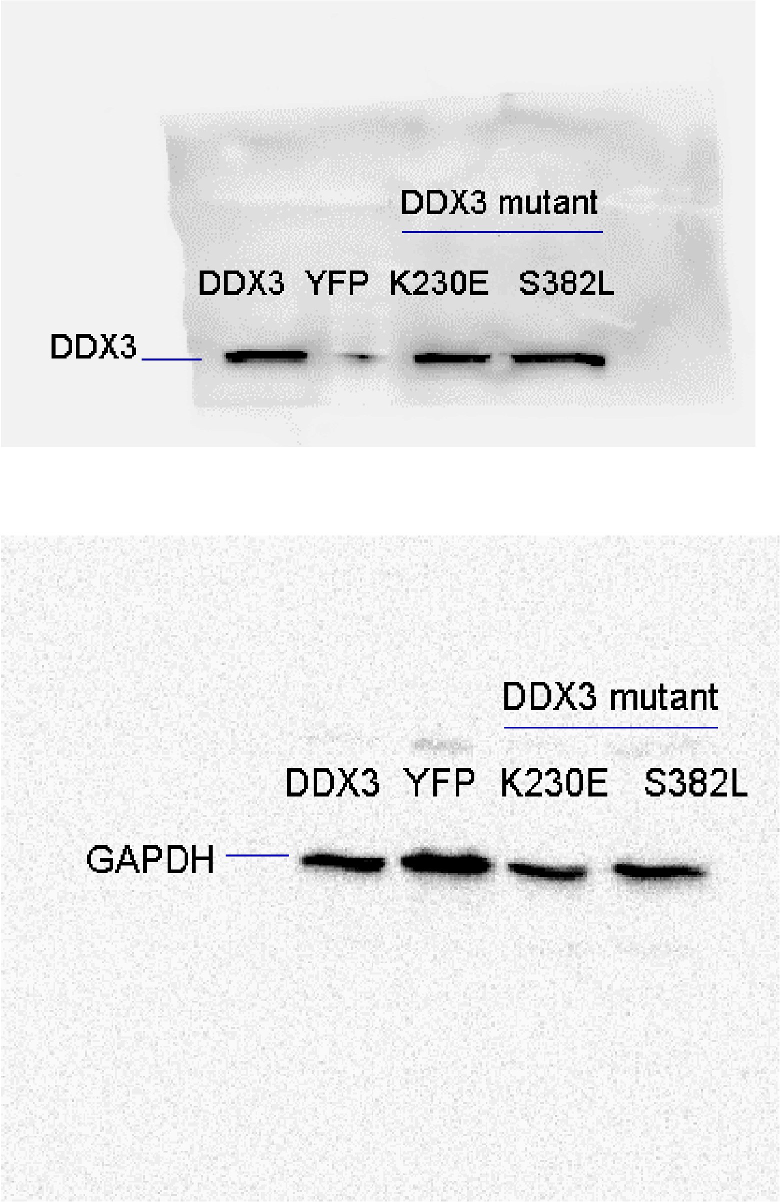
DDX3 mutant
DDX3 YFP K230E S382L
DDX3
DDX3 mutant
DDX3 YFP K230E S382L
GAPDH
